# Added Value of Using a Cocktail of F-18 Sodium Fluoride and F-18 Fluorodeoxyglucose in Positron Emission Tomography/Computed Tomography for Detecting Bony Metastasis: A Case Report

**DOI:** 10.1097/MD.0000000000000687

**Published:** 2015-04-03

**Authors:** Hung-Pin Chan, Chin Hu, Chang-Ching Yu, Tsung-Chi Huang, Nan-Jing Peng

**Affiliations:** From the Department of Nuclear Medicine (H-PC, CH, C-CY, T-CH, N-JP), Kaohsiung Veterans General Hospital; Department of Information Engineering (C-CY), I-Shou University, Kaohsiung; and National Yang-Ming University (N-JP), School of Medicine, Taipei, Taiwan.

## Abstract

Current nuclear imaging of the skeletal system is achieved using technetium-99m (Tc-99m) methylene diphosphonate (MDP), F-18 sodium fluoride (NaF), or F-18 fluorodeoxyglucose (FDG). However, comparisons of these are rare in the literature.

We present a case of a 51-year-old female with suspicious lung cancer due to main symptoms of dyspnea, nonproductive cough, and pleural pain. Tc-99m MDP whole-body bone scan (WBBS) showed multiple bony metastases. Five days later, positron emission tomography/computed tomography (PET/CT) images using both F-18 NaF and a cocktail of F-18 NaF and F-18 FDG were obtained on the same day 2 hours apart. The former showed more foci and precisely showed bony lesions compared to those obtained using Tc-99m MDP WBBS. However, the latter demonstrated more extensive radiotracer uptake, especially in osteolytic lesions, and additional soft tissue lesions in the left axillary and surpraclavicular nodes as well as the left pleura. Surgical biopsy was performed in left axillary nodes, and the metastatic carcinoma was found to be of breast origin.

This case demonstrated that a cocktail of F-18 NaF and F-18 FDG could be useful in PET/CT for not only detecting more skeletal lesions but also guiding biopsies accurately to the affected tissue.

## INTRODUCTION

Technetium-99m (Tc-99m) methylene diphosphonate (MDP) whole-body bone scan (WBBS) is widely used around the world and highly sensitive and cost-effective for bone metastasis screening and malignant disease follow-up after treatment. F-18 sodium fluoride (NaF) positron emission tomography (PET) is more sensitive than Tc-99m MDP WBBS in detecting bony metastasis.^1–3^ With the help of computed tomography (CT), F-18 NaF PET/CT leads to superior sensitivity and specificity in breast cancer patients with osteosclerotic bone metastases.^4^ F-18 fluorodeoxyglucose (FDG) PET could complement F-18 NaF or Tc-99m MDP WBBS in detecting bone metastasis.^5^ In general, a combination of these techniques may allow for improved imaging in equivocal bone lesions and earlier detection of bone metastasis. We present a case of multiple bony metastases of breast origin and demonstrate the added value of combining F-18 NaF and F-18 FDG in PET/CT, comparing it with Tc-99m MDP WBBS and F-18 NaF PET/CT.

## CONSENT

Written informed consent was obtained from the patient in this case report, and we have permission to use the accompanying images.

## CASE REPORT

**FIGURE 1:**
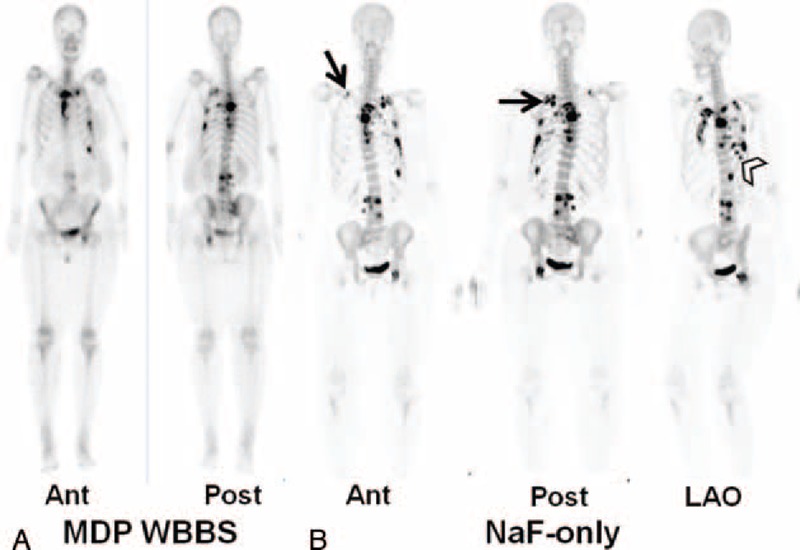
(A) Technetium-99m (Tc-99m) methylene diphosphonate (MDP) whole-body bone scan (WBBS) showed hot MDP uptake in the sternum, left side of the rib cage, thoracic and lumbar regions of the spine, right sacroiliac joint, and left ischium, suggesting multiple bony metastases. (B) Maximum intensity projection of F-18 NaF PET/CT at anterior, posterior, and left anterior oblique views showed new foci at 1st right and 2nd left posterior ribs (arrows) and lateral 7th to 9th ribs (arrow head) and more precisely showed bony lesions than seen using Tc-99m MDP WBBS. NaF = sodium fluoride, PET/CT = positron emission tomography/computed tomography.

**FIGURE 2:**
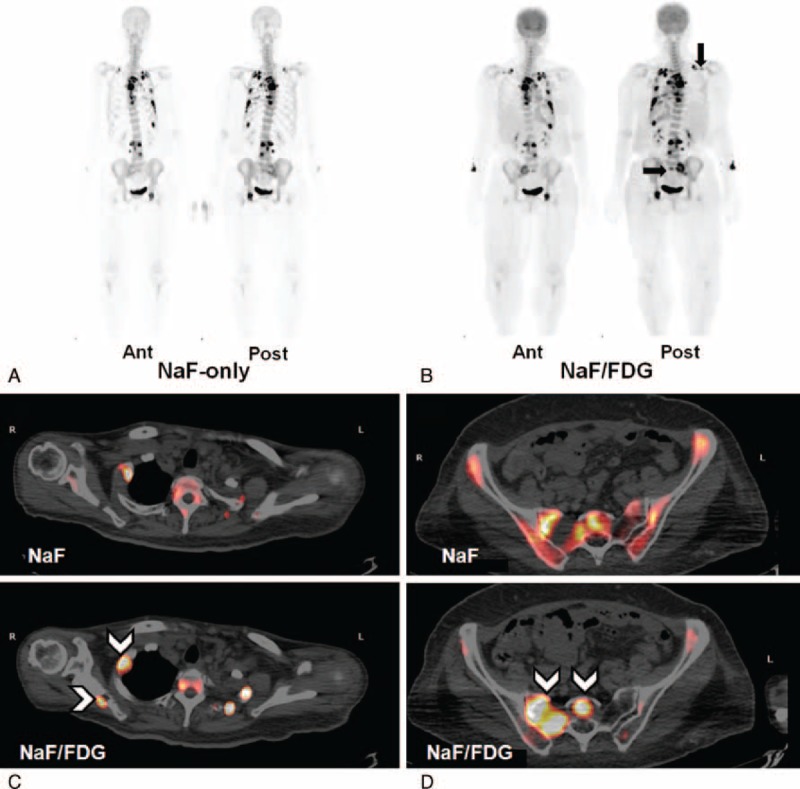
(A) and (B) Maximum intensity projection of F-18 NaF PET/CT and F-18 NaF/FDG PET/CT at anterior and posterior views. The latter radiotracer reveals new foci in the right scapula and sacrum (black arrows), and more extensive uptake in lesions than the former. (C) and (D) Transaxial views of F-18 NaF/FDG PET/CT images show new foci in the right scapula and sacrum, and more extensive radiotracer uptake in the osteolytic portion of the 1st right rib and right sacroiliac region, compared to images using F-18 NaF only (arrow heads). FDG = fluorodeoxyglucose, NaF = sodium fluoride, PET/CT = positron emission tomography/computed tomography.

**FIGURE 3:**
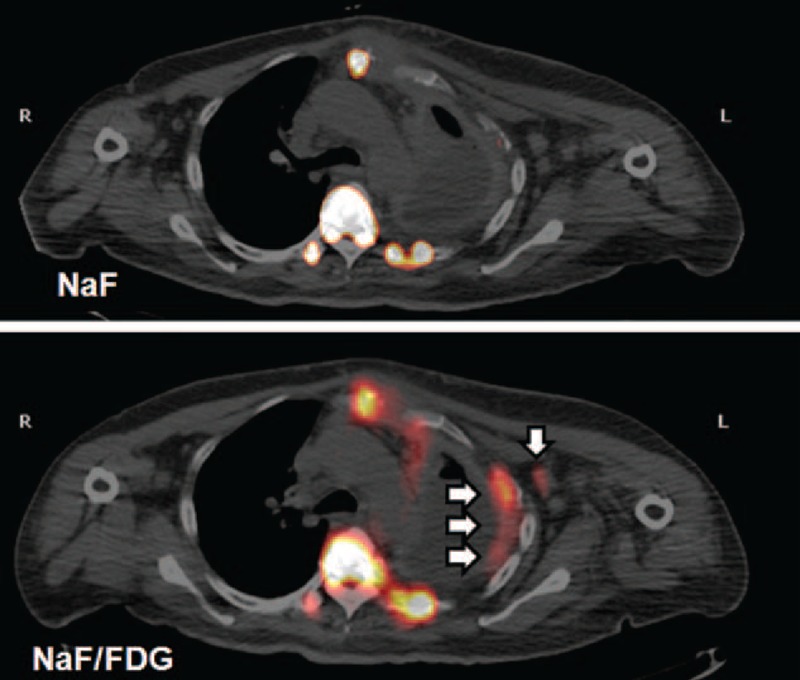
A cocktail of F-18 NaF/FDG revealed soft tissue uptake in left axillary nodes and left pleura (white arrows) and surpraclavicular nodes. Surgical biopsy was performed in the left axillary nodes 2 days later according to these findings. Pathology revealed poorly differentiated adenocarcinoma of breast origin. FDG = fluorodeoxyglucose, NaF = sodium fluoride.

A 51-year-old woman had a nodule of unknown pathology excised from her left breast 9 years before presenting to our clinic. She was admitted under the impression of suspected lung cancer due to dyspnea, nonproductive cough, pleural pain, poor appetite, swelling in all limbs, and recent weight loss. Tc-99m MDP WBBS showed hot MDP uptake in the sternum, left side of the rib cage, thoracic and lumbar regions of the spine, right sacroiliac joint, and left ischium, suggesting multiple bony metastases (Figure 1A). Five days later, PET/CT was performed after administering an F-18 NaF/FDG cocktail. F-18 NaF PET/CT showed new foci at the 1st right and 2nd left posterior ribs and lateral 7th to 9th ribs. It more precisely revealed obvious bony lesions than did the Tc-99m MDP WBBS (Figure 1B). It also revealed new foci in right scapula and sacrum and showed more extensive radiotracer uptake in lesions than did PET/CT using NaF only (Figure 2). In addition, it revealed soft tissue uptake in left axillary nodes, left pleura, and surpraclavicular nodes. Surgical biopsy was performed in the left axillary nodes 2 days later according to these findings (Figure 3). Pathology revealed poorly differentiated adenocarcinoma of breast origin. Therefore, the tentative diagnosis was cancer in the left breast with regional lymph node invasion, bony metastases, and pleural seeding on the left side (cT4N3M1, stage IV). Chemotherapy with Taxol (Corden Pharma Latina S.p.A.) was initiated, and she was discharged in stable condition.

One month later, the patient was readmitted because of progressive dyspnea and massive left-sided pleural effusion. A pigtail catheter was inserted to drain the effusion and a thoracoscope was used for decortication because of complicated emphysema. She was then under regular follow-up at our outpatient department (OPD) where she was given Xeloda (Productos Roche, S.A. de C.V.) for 6 months and Tamoxifen (AstraZeneca UK Limited) for 12 months. Zometa (Novartis Pharma Stein AG) and Xgeva (GlaxoSmithKline, Amgen Manufacturing, Limited) were also given for bone metastasis.

After regular follow-up in our OPD for 4 years, she was hospitalized again. Abdominal CT showed progressive changes in the pleural seeding and diffuse liver and retroperitoneal metastasis. She expired as a result of respiratory failure.

## DISCUSSION

Tc-99m MDP WBBS is a highly sensitive and cost-effective method of nuclear imaging of the skeletal system and is widely used around the world. However, its poor spatial resolution in planar scintigraphy limits its overall usefulness.^6^ Single-photon emission computed tomography (SPECT)/CT could provide added value in assessing suspected bone metastasis when compared to scintigraphy alone and CT.^7^ However, it is less sensitive than F-18 NaF PET/CT in detecting bony metastasis.^1–4^ Similar to Tc-99m MDP, F-18 NaF accumulates primarily in osteoblastic lesions of bone metastasis. In this case, we found hot foci in the same location as that shown on WBBS and F-18 NaF PET/CT, revealing osteolytic lesions in the CT component of the PET/CT. Tarnawska-Pierscinska et al^8^ demonstrated increased accumulation of F-18 NaF in metastatic foci of both osteoblastic and osteolytic lesions. Each osteolytic lesion is accompanied by low osteoblastic activity, which can be observed in F-18 NaF PET images, but may not be observed in Tc-99m MDP SPECT images. In our case, these osteolytic lesions, accompanied by osteoblastic activity of bone metastasis, were confirmed by the F-18 NaF PET/CT image.

As described in the previous articles, F-18 NaF and Tc-99m MDP are useful for characterizing osteoblastic bone lesions, whereas F-18 FDG is more sensitive in detecting osteolytic lesions.^8–9^ A recent international multicenter trial compared the performance of F-18 NaF/FDG PET/CT with F-18 NaF alone and F-18 FDG alone in various cancer patients, performing 3 PET/CT scans sequentially within 4 weeks for each patient. This trial demonstrated promising results with F-18 NaF/FDG PET/CT, making improved and less-expensive patient care a possibility.^5^ Another prospective trial compared F-18 FDG PET/CT with F-18 NaF/FDG PET/CT, injecting F-18 NaF subsequent to the initial F-18 FDG injection, doing so on the same day, citing patient convenience and reduced radiation exposure from the CT component. F-18 NaF/FDG-based images appeared to have greater detection sensitivity for osseous lesions than F-18 FDG-based images in this population.^10^ Moreover, Harisankar et al^11^ demonstrated that coinjection of F-18 NaF and F-18 FDG for PET/CT imaging in patients with breast cancer showed definite bone metastasis lesions with soft tissue uptake in metastatic lymph nodes. This study, however, lacks biopsy-proven metastasis in the lymph nodes. Our patient received F-18 FDG injection within 2 hours of the F-18 NaF injection, and 2 PET/CT scans were performed individually after each radiotracer injection. This protocol provided a comparison of the F-18 NaF and F-18 NaF/FDG PET/CT images. We found that the latter could be useful in not only detecting more skeletal lesions but also evaluating visceral lesions. We demonstrated the added value of a cocktail of F-18 NaF/FDG for PET/CT imaging to gain an accurate interpretation of the lesions in the skeleton and extraosseous findings, as compared to images obtained using F-18 FDG alone.

A combination of these images may allow for improved imaging of equivocal bone lesions or support for earlier detection of bone metastasis. In our patient, it showed fewer equivocal bone lesions, helping the physician to identify and characterize them. We demonstrated that a cocktail of F-18 NaF and F-18 FDG could be useful in PET/CT for not only detecting more skeletal lesions but also guiding biopsies accurately. It should be used in routine practice in the future once additional studies are completed and the best approach is determined.

## Acknowledgments

The authors would like to thank our department colleagues and the devotion of this patient.
